# Avacopan or Glucocorticoids for Severe Antineutrophil Cytoplasmic Autoantibody–Associated Rapidly Progressive Glomerulonephritis

**DOI:** 10.1016/j.ekir.2025.08.008

**Published:** 2025-08-13

**Authors:** Stanislas Faguer, Charlotte Gabilan, Magali Colombat, Alexis Cassard, Clément Kounde, Juliette Pellegrini, Antoine Huart, Julie Belliere, David Ribes

**Affiliations:** 1 Department of Nephrology and Organ Transplantation - Referral Center for Rare Kidney Diseases, University Hospital of Toulouse, Toulouse, France; 2 Faculty of Health, Toulouse University, Toulouse, France; 3 Institute of Metabolic and Cardiovascular Diseases, UMR 1297, National Institute for Health and Medical Research, Toulouse, France; 4 Department of Pathology, Toulouse University Institute of Cancer - Oncopole, Toulouse, France

**Keywords:** ANCA, avacopan, outcomes, rapidly progressive glomerulonephritis, vasculitis

## Abstract

**Introduction:**

The best immunosuppressive regimen to treat severe forms of antineutrophil cytoplasmic autoantibody (ANCA)-associated rapidly progressive glomerulonephritis (RPGN) remains elusive because patients with the lowest estimated glomerular filtration rate (eGFR) have been excluded from most studies, and kidney biopsy has not been a prerequisite for inclusion.

**Methods:**

In a cohort of 70 adult patients with severe ANCA-RPGN (eGFR: 0–30 ml/min per 1.73 m^2^ [mean: 12 ± 8]; anti-myeloperoxidase [MPO] antibodies: 76%, relapsing ANCA-associated vasculitis [AAV]: 6%) where a kidney biopsy was available, we compared kidney outcomes at month 6 and 12 according to induction regimen scheme (avacopan- vs. glucocorticoid [GC]-based).

**Results:**

Fifty patients received a GC regimen combined with cyclophosphamide (CYC) (30%), rituximab (RTX, 54%) or a combination of both (16%), whereas 20 patients received avacopan combined with RTX (90%) or RTX plus CYC (10%). Half of the patients in each group received plasma exchanges. The percentage of crescentic glomeruli (a reliable marker of kidney vasculitis activity) correlated with eGFR at presentation, whereas the percentage of glomerulosclerosis (a surrogate marker for chronic kidney lesions) correlated with maximal kidney improvement at month 6. After adjustment for histopathology, the induction regimen (RTX, CYC, or both) as well as the use of GCs or avacopan-based induction regimen were associated with similar kidney response at month 6, whatever end point was used. Reversible hepatitis requiring avacopan withdrawal occurred in 3 patients. No new safety signal was identified in the avacopan group.

**Conclusion:**

At months 6 and 12, patients with severe forms of ANCA-RPGN and receiving avacopan plus a short course of GCs have similar kidney recovery rates to patients receiving GC regimen.

## Graphical abstract

**Figure:**
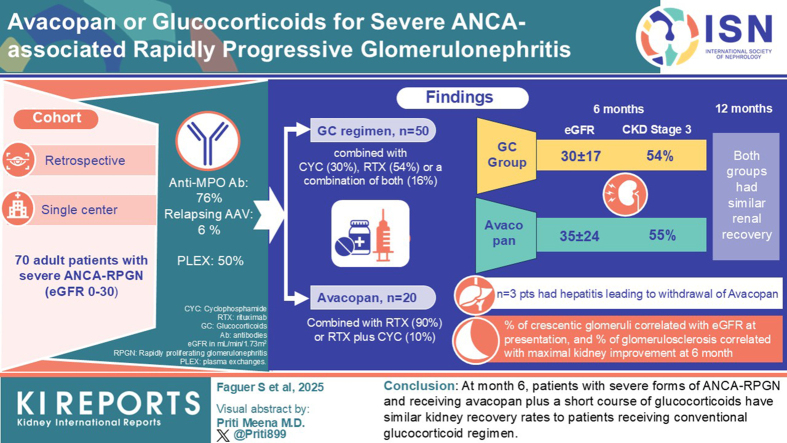

Severe ANCA-RPGN is associated with a high risk of death, kidney failure, and chronic kidney disease.^1^ Following a diagnosis of ANCA-RPGN, a kidney biopsy is routinely performed to rule out alternative diagnoses; to estimate kidney outcomes (by assessing chronicity and activity); and to adapt the treatment to optimize kidney response while reducing the risks related to immunosuppression.^2^, ^3^, ^4^, ^5^, ^6^, ^7^ A kidney biopsy can reveal a wide variety of lesions, including, among others, glomerular or arteriolar vasculitis, necrosis of the glomerular tuft, extracapillary crescents, Bowman’s capsule rupture, tubular necrosis, glomerulosclerosis, interstitial fibrosis, and tubulitis.^1^^,^^4^

When active ANCA-RPGN is suspected, several variables will affect the severity of kidney dysfunction at presentation, as well as renal improvement. These variables will be related to the following: (i) the patient (past history of diabetes mellitus, hypertension, or nephrotoxic exposure with subsequent kidney fibrosis; age; intrinsic sensitivity or resistance to kidney injury); or (ii) the vasculitis (MPO or proteinase-3 ANCA serotype; intensity and duration of renal vasculitis; and specific kidney response to vasculitis; e.g., thrombotic microangiopathy, granuloma, acute tubular necrosis). In addition, RPGN can occur during the first flare-up of AAV or during a relapse. Thus, previously fixed kidney lesions, whether they are related to AAV, can uncouple the severity of the kidney lesions relating to the current AAV flare-up (that can resolve with treatment) and the eGFR, precluding accurate prediction of renal improvement. Accordingly, analysis of kidney biopsies from patients with ANCA-RPGN has demonstrated that various pathological patterns (crescentic RPGN, sclerosed RPGN, or mixed pathology) are associated with similar renal severity at presentation (eGFR < 30 ml/min per 1.73 m^2^) but different kidney outcomes.^4^

Whereas kidney pathology correlates with the risk of kidney failure and with the best eGFR that can be reached at month 6 or 12 in ANCA-RPGN, most randomized controlled trials for AAV have included patients with a wide range of eGFRs at baseline (i.e., with highly variable potential for renal improvement); they have excluded patients with the most severe forms of ANCA-RPGN (i.e., eGFR < 15 ml/min per 1.73 m^2^) even though they were the most likely to benefit from new therapies; and they have rarely used kidney pathology to obtain a comprehensive view of kidney outcomes.^8^, ^9^, ^10^, ^11^ There are still uncertainties regarding the best immunosuppressive regimen in severe forms of RPGN. In addition, current renal risk scores were designed to identify patients who are more at risk of kidney failure or death, but none were designed to estimate the maximal eGFR improvement even though final eGFR at months 6 or 12 will predict long-term kidney failure as well as the risk of cardiovascular events.

The recent approval of avacopan, an oral C5a receptor inhibitor, for severe and active AAV offers new possibilities of therapeutic combinations.^2^ However, avacopan was mainly developed as a GC-sparing agent, and patients with very low eGFR (< 15 ml/min per 1.73 m^2^) were excluded from the trial.^8^ In the ADVOCATE phase 3 study, CYC or RTX were combined with GCs or with avacopan (in this latter group, GCs were withdrawn after less than 4 weeks). At month 6, the rate of AAV remission and eGFR were similar in both groups.^8^ In “real-life” retrospective studies, avacopan was associated with a favorable risk-benefit ratio; however, the dose of GCs was highly variable and the number of patients with very severe ANCA-RPGN was small, precluding firm conclusions.^12^, ^13^, ^14^, ^15^, ^16^

In this comprehensive study, we retrospectively reviewed the kidney outcomes of 70 patients with AAV with severe ANCA-RPGN (eGFR: 0–30 ml/min per 1.73 m^2^) and available kidney biopsies. The efficacy of GC-based and avacopan-based regimens was compared at months 6 and 12.

## Methods

### Patients

To be included in this retrospective study, patients had to fulfill the following criteria: (i) a diagnosis of AAV according to the American College of Rheumatology and the European League Against Rheumatism criteria with positive anti-MPO or anti–proteinase-3 ANCA, (ii) active AAV with a Birmingham Vasculitis Activity Score > 3, (iii) a diagnosis of RPGN with kidney biopsy confirming the diagnosis of AAV-associated pauci-immune glomerulonephritis, and (iv) a minimum follow-up period of 6 months. All patients were followed up with in the Department of Nephrology and Organ Transplantation at the University Hospital of Toulouse (France) between 2009 and 2024. Only patients aged ≥ 18 years were included.

Patients in the GCs group were referred between 2009 and 2020 (first flare), whereas patients in the avacopan group were referred between 2021 and 2024 (first flare or relapse).

### Immunosuppressive Regimen

The induction regimen included RTX (375 mg/m^2^/wk for 4 weeks), i.v. CYC (500–750 mg/m^2^ depending on the patient's eGFR and age; 1 infusion on day 0, then again on day 15 and day 30, and then 3 additional infusions 3 weeks apart), and a combination of RTX (375 mg/m^2^/wk for 4 weeks) and CYC (500 mg/m^2^ on day 1 and day 15). In patients receiving apheresis, 7 sessions were performed in 14 days (plasma exchange or double filtration plasmapheresis).

In patients receiving a GC-based regimen, doses of GCs followed a high-dose scheme (i.e., doses were similar to those received by patients randomized in the “high-dose GC” group in the PEXIVAS study, except for 3 who received a “PEXIVAS low-dose” protocol^17^). In patients receiving avacopan, clinicians were encouraged to stop GCs in the 4 weeks following the start of avacopan; however, the duration varied according to the response to treatment.

### Clinical and Pathological Data

Clinical data included demographic profile and routine clinical and laboratory findings that were obtained from medical records. In patients requiring dialysis at baseline, eGFR was considered as 0 ml/min per 1.73 m^2^.

Processing of kidney biopsies included optical microscopy and immunofluorescence. For optical microscopy, all samples were stained with hematoxylin and eosin, periodic acid-Schiff, Masson’s trichrome, and Jones methenamine silver. For immunofluorescence, 0.3-μm cryostat sections were stained with polyclonal antibodies to IgG, IgM, IgA, C3, C1q, kappa, lambda, fibrinogen and albumin-FITC (Agilent polyclonal rabbit). The percentages of sclerosed glomeruli (sclerosed/total glomeruli), crescentic glomeruli (crescentic/total glomeruli) and interstitial fibrosis / tubular atrophy (IF/TA) areas were noted.

### Kidney Outcomes

To comprehensively analyze the interplay between kidney pathology, immunosuppressive regimen, and response to treatment, we used multiple readouts of kidney outcome: a nominal value for eGFR at month 6, the difference between baseline and month 6 eGFRs, the percentage of patients with eGFR ≥ 30 ml/min per 1.73 m^2^ (estimated using the Chronic Kidney Disease-Epidemiology Collaboration formula) at month 6; and the percentage of patients requiring chronic dialysis at month 6. Primary outcome was the eGFR at month 6 in each group. Secondary outcomes included other readouts of kidney outcomes, the relationship between kidney biopsy (glomerulosclerosis, percentage of crescentic glomeruli, IF/TA) and eGFR at month 6, eGFR at month 12, and safety.

### Statistical Analyses

Continuous variables were expressed as mean and SD, and compared with the Mann-Whitney U test or the Kruskal-Wallis test, as appropriate. Discontinuous variables were expressed as numbers and percentages and compared using Fisher exact test. Multivariable analyses were performed using logistic regression after inclusion of variables significantly associated with the outcome by univariate analyses (*P* < 0.05). A maximum of 4 variables were included in the models and 4 models were tested. Comparison of eGFR trajectories according to immunosuppressive regimen was performed using mixed effect analysis. A *P*-value < 0.05 was considered as significant.

### Ethics

This study was conducted according to the Helsinki Declaration, as revised in 2004. The Institutional Review Board of the University Hospital of Toulouse authorized the retrospective collection of data from patients with AAV followed up with at the University Hospital of Toulouse (agreement number RnIPH 2023-37 and RnIPH 2024-009). In accordance with its recommendations, written informed consent was waived. All patients who received avacopan were included in the biological samples collection Nephrogene 2.0, a collection approved by the national French Ethical Committee (RC31/21/0154; clinical trials NCT05318196).

## Results

### Characteristics of the Patients

**Figure 1:**
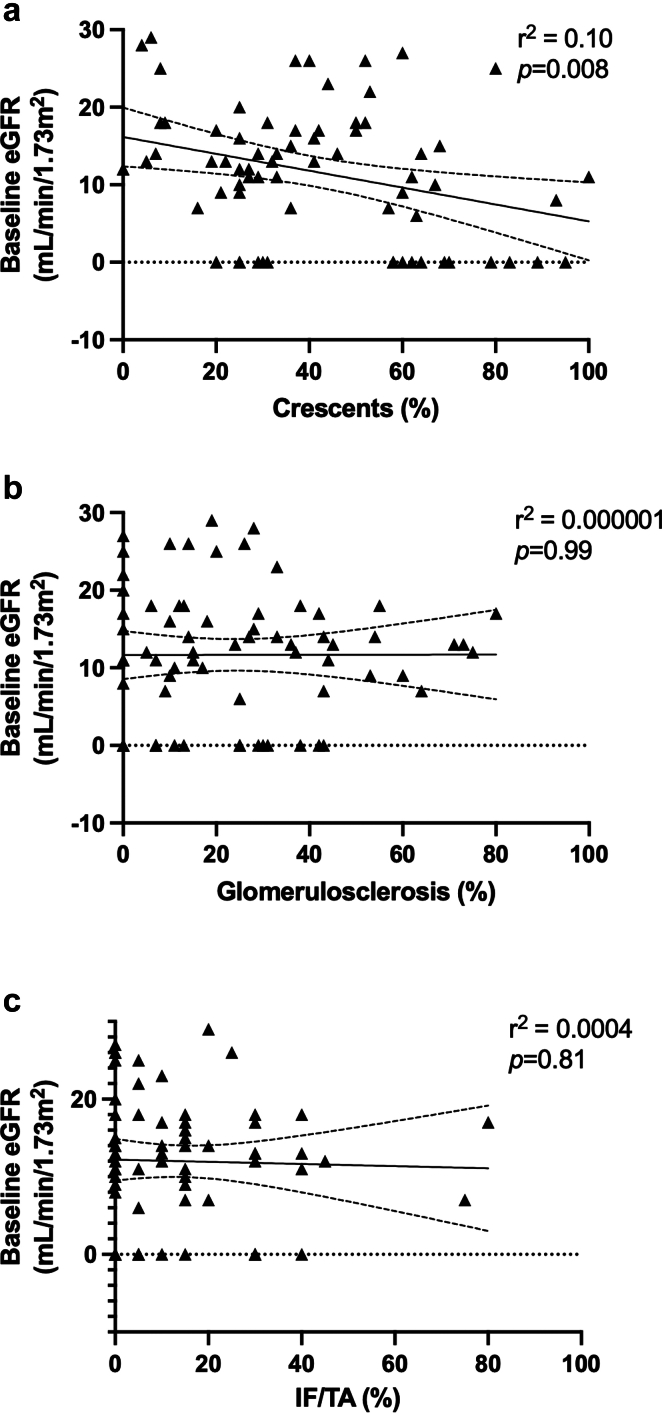
Correlation between eGFR at baseline and anomalies on kidney biopsy. eGFR, estimated glomerular filtration rate; IF/TA, interstitial fibrosis/tubular atrophy.

Seventy patients with active ANCA-RPGN and eGFR < 30 ml/min per 1.73 m^2^ at baseline fulfilled the inclusion criteria (male: 59%, mean age: 67 ± 15 years) (Table 1). A majority of patients had anti-MPO antibodies (76%). On admission, mean eGFR was 12 ± 8 ml/min per 1.73 m^2^ and 17 patients (24%) required kidney replacement therapy. Kidney biopsies showed a large range of the following: IF/TA (from 0%–80% of the kidney slice), glomerulosclerosis (0%–80%), and crescentic glomeruli (0%–100%) (Figure 1). eGFR on admission correlated with the proportion of crescentic glomeruli (r^2^ = 0.10, *P* = 0.008) but not with the extent of glomerulosclerosis or IF/TA. Granuloma and thrombotic microangiopathy were observed in 1 and 3 patients, respectively.

**Table 1:** Characteristics of patients

Characteristics	Overall cohort *N* = 70	Induction regimen		*P*-value
		GCs *n* = 50	Avacopan *n* = 20	
Sex (male; *n*, %)	41 (59)	28 (56)	13 (65)	0.59
Age (yrs; mean ± SD)	67 ± 15	69 ± 12	61 ± 19	0.14
Vasculitis parameters				
ANCA antibodies (*n*, %)				
Anti-MPO	53 (76)	38 (76)	15 (75)	1.00
Anti-PR3	47 (24)	12 (24)	5 (25)	
AAV new onset	4 (6)	0	4 (20)	< 0.01
Organ involvements				
Lung	30 (43)	21 (42)	9 (45)	1.00
Heart	4 (6)	3 (6)	1 (5)	1.00
ENT	16 (23)	12 (24)	4 (20)	1.00
Nerves	13 (19)	10 (20)	3 (15)	0.75
Purpura	2 (3)	0	2 (10)	0.08
BVAS (mean ± SD)	18 ± 6	18 ± 5	17 ± 5	0.49
Kidney parameters at baseline				
eGFR (ml/min per 1.73 m^2^; mean ± SD)	12 ± 8	11 ± 8	13 ± 9	0.45
eGFR (*n*, %)				1.00
0–14 ml/min per 1.73 m^2^	47 (67)	34 (68)	13 (65)	
15–29 ml/min per 1.73 m^2^	23 (33)	16 (32)	7 (35)	
uPCr (g/g; mean ± SD)	1.9 ± 1.8	2 ± 1.8	1.6 ± 1.9	0.19
Dialysis (*n*, %)	17 (24)	12 (24)	5 (25)	1.00
Kidney biopsy				
Glomerulosclerosis (%; mean ± SD)	23 ± 22	26 ± 22	16 ± 17	0.04
Crescents (%; mean ± SD)	39 ± 25	39 ± 27	39 ± 19	0.87
IF/TA (mean ± SD)	14 ± 16	15 ± 18	10 ± 10	0.25
Berden scoring				0.57
Focal	14 (20)	5 (25)	9 (18)	
Crescentic	27 (39)	7 (35)	20 (40)	
Mixed	20 (28)	7 (35)	13 (26)	
Sclerotic	9 (13)	1 (5)	8 (16)	
Induction regimen				0.004
Cyclophosphamide	15 (22)	15 (30)	0	
Rituximab	45 (64)	27 (54)	18 (90)	
Cyclophosphamide + Rituximab	10 (14)	8 (16)	2 (10)	
Plasma exchanges	38 (54)	28 (56)	10 (50)	0.79
Kidney outcomes (month 6)				
eGFR (ml/min per 1.73 m^2^; mean ± SD)	32 ± 19	30 ± 17	35 ± 24	0.58
eGFR (*n*, %)				0.90
0–14 ml/min per 1.73 m^2^	9 (13)	7 (14)	2 (10)	
15–29 ml/min per 1.73 m^2^	23 (33)	16 (32)	7 (35)	
30–59 ml/min per 1.73 m^2^	33 (47)	24 (48)	9 (45)	
60 ml/min per 1.73 m^2^	5 (7)	3 (6)	2 (10)	
Dialysis (*n*, %)	5 (7)	3 (6)	2 (10)	0.64
eGFR change (ml/min per 1.73 m^2^; mean ± SD)	20 ±18	19 ± 15	23 ± 23	0.92
Kidney outcomes (month 12; *n* = 64)				
eGFR (ml/min per 1.73 m^2^; mean ± SD)	33 ± 19	30 ± 17	40 ± 25	0.28
eGFR (*n*, %)				0.77
0–14 ml/min per 1.73 m^2^	8 (12)	7 (14)	1 (7)	
15–29 ml/min per 1.73 m^2^	25 (39)	20 (40)	5 (36)	
30–59 ml/min per 1.73 m^2^	26 (41)	20 (40)	6 (43)	
60 ml/min per 1.73 m^2^	5 (8)	3 (6)	2 (14)	
Dialysis (*n*, %)	6 (9)	6 (12)	0	0.32
eGFR change (ml/min per 1.73 m^2^; mean ± SD)	20 ± 17	19 ± 16	24 ± 21	0.44

AAV, ANCA-associated vasculitis; ANCA, antineutrophil cytoplasmic autoantibodies; BVAS, Birmingham Vasculitis Activity Score; eGFR; estimated glomerular filtration rate; ENT, ear, nose, throat; GCs, glucocorticoids; IF/TA, interstitial fibrosis/tubular atrophy; MPO, myeloperoxidase; PR3, proteinase-3; uPCr, urinary protein-to-creatinine ratio.

Avacopan and GC patients had similar characteristics (age, sex, ANCA antibodies, organ involvements, and kidney parameters at baseline), except for the percentage of sclerosed glomeruli on kidney biopsy which was slightly higher in the GC group (16 ± 17 vs. 26 ± 22, *P* = 0.04) and the induction regimen (Table 1). GC patients (*n* = 50) received i.v. CYC (30%), RTX (54%), or a combination of both (16%); whereas avacopan patients (*n* = 20) received RTX (90%) or RTX plus CYC (10%) (*P* = 0.004). Half the patients received apheresis in both groups despite the lack of specific matching. In avacopan patients, the duration of GC intake was 1.9 ± 1.8 months (range: 0–6) versus 15 ± 12 months in GCs patients (range: 3–40) (*P* < 0.0001). Thereafter, the doses of GCs received by patients in the GCs and avacopan groups were 4330 ± 920 mg compared with 1745 ± 1088 mg (*P* < 0.0001), respectively, including pulses (1500–2500 mg) in 18 (36%) compared with 3 (15%).

In GC patients (mean follow-up: 56 ± 35 months), the maintenance regimen relied on RTX (*n* = 33, 66%), azathioprine (*n* = 8, 16%) or mycophenolic acid (*n* = 1, 2%). Eight patients did not receive maintenance. In contrast, all patients in the avacopan group (mean follow-up: 15 ± 9 months) received maintenance RTX.

### Comprehensive Analysis of Kidney Outcomes

**Figure 2:**
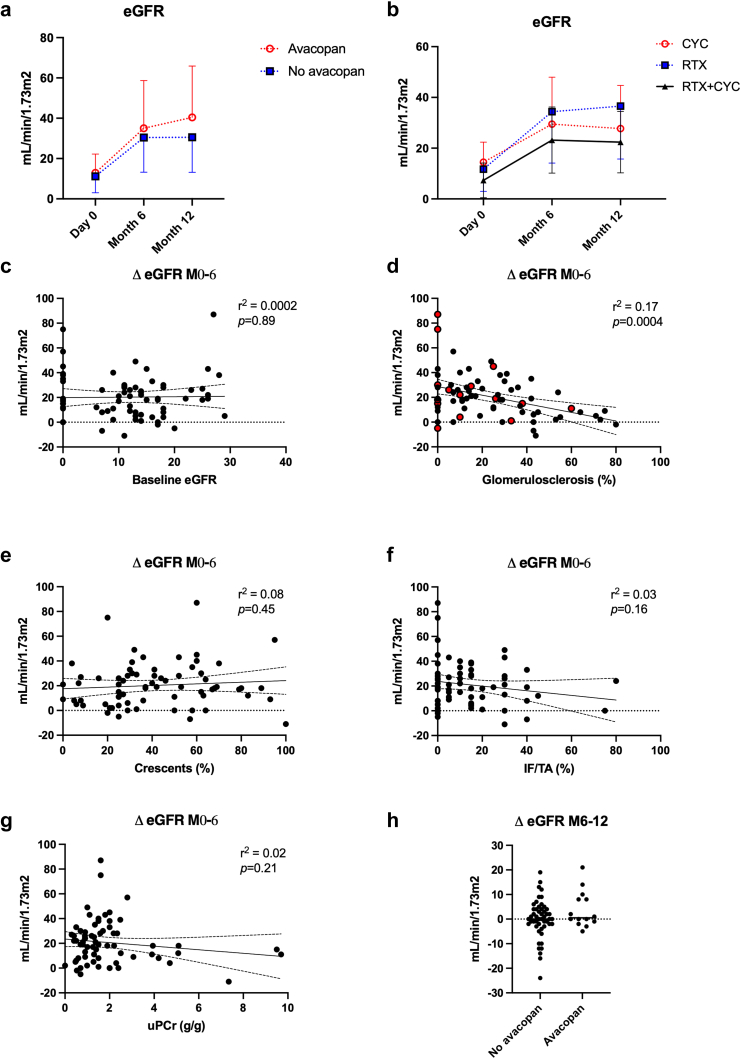
Correlation between kidney outcomes and characteristics at baseline. In Panel B, all patients were included in the analyses. In Panel D, red and black circles represent patients who received or not avacopan, respectively. Δ eGFR M0-6, eGFR at month 6 minus eGFR at baseline; Δ eGFR M6-12, eGFR at month 12 minus eGFR at month 6; CYC, cyclophosphamide; eGFR, estimated glomerular filtration rate; IF/TA, interstitial fibrosis / tubular atrophy; RTX, rituximab; uPCr, urinary protein- to- creatinine ratio.

At month 6, patients receiving GC-based and avacopan-based induction regimens had similar eGFR (30 ± 17 vs. 35 ± 24 ml/min per 1.73 m^2^, respectively) and similar nominal increase in eGFR (+19 ± 15 vs. +23 ± 23 ml/min per 1.73 m^2^) (Figure 2). Four patients had reached kidney failure and required chronic dialysis (2 in each group, no significant difference). With similar eGFR on admission, the increase in eGFR at month 6 was highly variable. For example, patients who required dialysis on admission had an increase in eGFR ranging from 0 to 80 ml/min per 1.73 m^2^. The same variability was observed when plotting eGFR increase for chronic (glomerulosclerosis, IF/TA) or active (crescent) lesions on kidney biopsy.

The number of patients who reached an eGFR ≥ 30 ml/min per 1.73 m^2^ (i.e., Chronic Kidney Disease Kidney Disease: Improving Global Outcomes stage 3 or less) was similar in both groups (54% and 55%). Trajectories for eGFR were similar whatever the induction regimen (RTX vs. CYC vs. RTX + CYC) and use of avacopan- or GCs-based regimens (Figure 2).

By multivariable analysis, the percentage of glomerulosclerosis on kidney biopsy and the need for dialysis at baseline significantly correlated with the probability of an increase in eGFR beyond 30 ml/min per 1.73 m^2^ at month 6. The association between anti-MPO ANCA and eGFR was close to significance (*P* = 0.05). When Berden scoring was included in the multivariable analyses (Table 2), eGFR at baseline, ANCA type, and histological pattern correlated with better eGFR at month 6 but neither the induction regimen nor avacopan or GCs were associated with kidney outcome.

**Table 2:** Predictive factors for eGFR ≥ 30 ml/min per 1.73 m^2^ at month 6 (multivariable analyses using logistic regression)

Variables	Model 1		Model 2		Model 3		Model 4	
	Odds ratios	*P*-value	Odds ratios	*P*-value	Odds ratios	*P*-value	Odds ratios	*P*-value
Baseline eGFR (per ml/min per 1.73 m^2^)	1.12 (1.03–1.23)	0.03	1.09 (1.01–1.17)	0.02	1.13 (1.04–1.23)	0.003	1.08 (1.01–1.16)	0.04
Berden class (vs. sclerotic)								
Focal	144 (6.3–3290)	0.002	133 (6.4–2752)	0.002			104 (5.4–1969)	0.002
Crescentic	9.9 (0.9–110)	0.06	11.6 (1.2–114)	0.04			10.7 (1.1–104)	0.04
Mixed	10.9 (1–120)	0.05	12.3 (1.2–126)	0.04			10.6 (1.5–106)	0.04
Anti-MPO ANCA (vs. Anti-PR3)	0.15 (0.03–0.76)	0.02	-	-	0.23 (0.05–1.03)	0.06	-	-
Glucocorticoids (vs. avacopan)	-	-	1.90 (0.51–7.01)	0.34	1.7 (0.47–6.2)	0.42	-	-
Induction regimen (vs. RTX)								
CYC							1.6 (0.23–11.4)	0.63
CYC + RTX							1.35 (0.27–6.7)	0.71

ANCA, anti-neutrophil cytoplasmic autoantibodies; CYC, cyclophosphamide; eGFR, estimated glomerular filtration rate; MPO, myeloperoxidase; PR3, proteinase-3; RTX, rituximab.

Finally, eGFR was available at month 12 for 64 patients. As shown in Figure 2, the mean difference between eGFR at months 6 and 12 was similar in avacopan-based and GCs-based regimens but interindividual heterogeneity was high. Nominal eGFR at month 12 was similar in both groups; however, a significant proportion of patients had frank eGFR decrease between months 6 and 12 in the GC group despite low rate of relapse in the first 12 months of follow-up (0/20 in the avacopan group and 2/50 in the GC group). Predictive factors of eGFR > 30 ml/min per 1.73 m^2^ at month 12 are shown in Supplementary Table S1.

### Extra Kidney Outcomes

After adjustment for age, Cox regression for survival analysis reported similar overall survival in patients receiving an avacopan- or GCs-based regimen. AAV was considered still active in 2 patients at month 6 (1 in each group).

### Tolerance of Avacopan

Among the 20 patients who received avacopan, hepatitis led to a withdrawal of avacopan in 3 cases (15%; period of exposure: 2–3 months). GCs were resumed in one of those cases (5 mg/d). Liver tests normalized thereafter (<1 month).

In 1 patient, both GCs and avacopan were stopped because of recurrent infections in a context of profound underlying immunosuppression (with a history of chronic lymphocytic leukemia, which was in complete remission at that time). One additional patient with a known history of bronchiectasis developed bacterial pneumonia requiring hospitalization and transient oxygen support.

## Discussion

In this study that included 70 patients with active ANCA-RPGN, with an eGFR < 30 ml/min per 1.73 m^2^ and where kidney biopsies were available, eGFR at presentation mainly correlated with markers of kidney vasculitis (percentage of crescentic glomeruli) whereas maximal kidney improvement at month 6 correlated with kidney disease chronicity (percentage of glomerulosclerosis). The induction regimen (CYC, RTX, or CYC plus RTX) as well as the use of GC-based or avacopan-based regimens were associated with similar kidney response at month 6, whatever end point was used (nominal eGFR, eGFR gain, eGFR ≥ 30 ml/min per 1.73 m^2^).

The correlation between kidney pathology and the long-term risk of kidney failure has been well-documented.^3^, ^4^, ^5^, ^6^, ^7^ Markers of chronicity (sclerotic pattern) were significantly associated with long-term kidney survival in the seminal paper by Berden *et al.*^4^ However, in randomized controlled trials that compared RTX (± CYC) to CYC,^9^^,^^11^ and high versus low-dose GC regimens^10^ for AAV, kidney biopsy was not required or not available for comparative analyses. In these studies, eGFR ranged from 15 to > 60 ml/min per 1.73 m^2^, a window associated with very high heterogeneity of kidney vasculitis severity, chronicity index, and predominant kidney pathology pattern. The same pitfalls were identified in the PEXIVAS and ADVOCATE studies that tested the efficacy of plasma exchanges or avacopan in unselected AAV patients.^8^^,^^10^

In the randomized MEPEX study, in which only patients with the most severe forms of ANCA-RPGN (serum creatinine > 500 μmol/l, roughly synonymous with an eGFR < 15 ml/min per 1.73 m^2^) were included, kidney biopsy was a prerequisite and the percentage of active or chronic lesions was well-balanced between the 2 groups of treatment giving statistical power to the positive effects of plasma exchanges in this setting.^18^ As shown in our series and in MEPEX, the percentage of glomerulosclerosis can range from 0% to 80% in patients with eGFR < 15 ml/min per 1.73 m^2^, a finding that highlights how an accurate comparison of kidney responses to therapies requires adjustment for kidney biopsies in patients with ANCA-RPGN. Therefore, after adjusting for the percentage of glomerulosclerosis, the initial eGFR did not correlate with eGFR at month 6, and kidney improvement was similar in GC and avacopan patients.

In addition, our findings highlight high interindividual heterogeneity in kidney recovery at month 6, and in eGFR changes from month 6 to 12. As already reported in the PEXIVAS trial,^10^ eGFR at month 6 strongly correlated with eGFR at month 12, suggesting that an early (i.e., before the development of irreversible lesions) and intensive regimen (allowing immediate prevention of new active lesions or transition toward chronic lesions) is required to optimize short- and long-term kidney outcomes. For example, there are still controversies regarding the use of plasma exchanges in patients with the most severe forms of ANCA-RPGN because the large nonadjusted PEXIVAS study showed no benefit from adding this treatment.^10^ However, a reanalysis of the trial results recently showed that plasma exchanges may improve early kidney recovery in severe forms of ANCA-RPGN.^17^ Although it is still unclear whether any beneficial effects can be linked to specific underlying kidney patterns in this study, the benefit of plasma exchanges was shown in a large retrospective cohort of 425 patients with ANCA-RPGN and clustered according to a histopathological score.^3^

The development of new drugs to control systemic AAV (avacopan, obinutuzumab, and maybe CAR-T cells) or the RPGN itself (pioglitazone NCT05946564, anti-claudin-1 NCT06047171) acts as a prompt to better categorize patients into clusters according to their underlying pathological or molecular kidney patterns, and their potential for kidney recovery. Although this will be challenging and will require large cohorts of patients, clustering of ANCA-RPGN will help to optimize and individualize treatment by indicating potential new combinations. For example, rather than opposing avacopan and GCs, the REVERSE randomized controlled trial (EU-CT 2024-519620-24-00) will compare kidney recovery at months 6 and 12 in patients with severe ANCA-RPGN receiving a standard-of-care immunosuppressive regimen plus GCs or avacopan+GCs. This design is in line with data from real-world observational studies from the US and Germany that show that GCs are frequently used beyond the 4 weeks formerly recommended in the ADVOCATE study.^8^^,^^13^^,^^19^

Given its retrospective design, our study has several biases, including the exclusion of patients without data available at month 6 (those who had died or been lost to follow-up). In addition, eGFR was not available for all patients at month 12. Nevertheless, we were able to include 70 patients with homogeneous kidney presentations and with an available kidney biopsy; however, the size of the study did not allow us to cluster patients in any profound way based on specific pathologic findings. Immunosuppressive regimens were not standardized; however previous large randomized controlled trials reported similar recovery with RTX, CYC, or RTX+CYC regimens.^9^^,^^11^ Furthermore, focusing our main analyses on month 6 allowed us to mostly circumvent the risk of bias relating to the heterogeneity of maintenance regimens. Lastly, the inclusion period of patients (before 2021 for 47/50 patients in the GC group and after 2021 for 20/20 patients in the avacopan group) may have biased results. However, the main characteristics were well-balanced at baseline between the 2 groups, except for the number of relapsing AAV that was higher in the avacopan group and may have put the avacopan group at a disadvantage (Table 1).

In summary, our study highlights how underlying kidney pathology can influence the best possible kidney response in severe ANCA-RPGN. At month 6, patients receiving avacopan and transient or low doses of GCs have similar kidney outcomes to patients who received an induction regimen with conventional doses of GCs. Adjustment of kidney response on a baseline kidney pathology score is needed to identify the best immunosuppressive combination for severe ANCA-RPGN.

## Disclosure

SF received consulting and SAB fees from Novartis, Abionyx Pharma, CSL-Vifor, GSK, and Alexion. DR received SAB fees from Novartis. All the other authors declared no competing interests.

## Supplementary Material

Supplementary File (PDF) **Table S1.** Predictive factors of eGFR ≥ 30 ml/min per 1.73 m^2^ at month 12 (multivariable analyses using logistic regression).
