# Supplementary material for: Avacopan or Glucocorticoids for Severe Antineutrophil Cytoplasmic Autoantibody–Associated Rapidly Progressive Glomerulonephritis
Source: Kidney Int Rep. 2025 Aug 13;10(11):3937–44. doi: 10.1016/j.ekir.2025.08.008 (PMC12639830; doi:10.1016/j.ekir.2025.08.008)
Supplement: Supplementary File (PDF) — Table S1. Predictive factors of eGFR ≥ 30 ml/min per 1.73 m2 at month 12 (multivariable analyses using logistic regression). [file mmc1.pdf]

Supplementary Table 1 : Predictive factors for eGFR ≥ 30 mL/min/m² at month 12 (multivariable analyses using logistic regression). eGFR, estimated glomerular filtration rate; ANCA, anti-neutrophil cytoplasmic antibodies; MPO, myeloperoxidase; RTX, rituximab; CYC, cyclophosphamide.

	**Model 1**		**Model 2**		**Model 3**	
**Variables**	**Odds ratios**	**P-value**	**Odds ratios**	**P-value**	**Odds ratios**	**P-value**
**Baseline eGFR (per mL/min/1.73m²)**	1.12 [1.03 – 1.23]	0.01	1.11 [1.02 – 1.21]	0.01	1.16 [1.05 – 1.29]	0.006
**Berden class (vs. Sclerotic)**						
**Focal**	21.4 [1.6 – 289]	0.02	13.8 [1.15 – 165]	0.04	158 [5.7 - 4395]	0.003
**Crescentic**	24.2 [2.1 – 281]	0.01	14.3 [1.37 - 150]	0.03	18.8 [1.35 - 261]	0.029
**Mixed**	6.1 [0.55 – 66.2]	0.14	5.6 [0.54 – 59.3]	0.15	9.8 [0.81 - 118]	0.07
**Anti-MPO ANCA (vs. Anti-PR3)**			0.38 [0.09 – 1.62]	0.01	0.15 [0.02 – 0.90]	0.04
**Glucocorticoids (vs. avacopan)**	1.61 [0.33 – 7.82]	0.56	1.07 [0.24 – 4.8]	0.93		
**Glucocorticoids pulses**	4.4 [1.1 – 17.8]	0.04			2.87 [0.61 – 13.4]	0.18
